# The human mycobiome: a critical yet understudied component of health and disease

**DOI:** 10.1099/mic.0.001631

**Published:** 2025-12-10

**Authors:** Rasoul Mohammadi, Hamid Morovati, Fatemeh Safari

**Affiliations:** 1 Department of Medical Parasitology and Mycology, School of Medicine, Infectious Diseases and Tropical Medicine Research Center, Isfahan University of Medical Sciences, Isfahan, Iran; 2 Department of Parasitology and Mycology, Faculty of Medicine, Tabriz University of Medical Sciences, Tabriz, Iran; 3 Department of Microbiology, Fal.C., Islamic Azad University, Isfahan, Iran

**Keywords:** disease association, fungal diversity, human mycobiome, microbial community

## Abstract

The human body hosts a complex and dynamic microbial community that is crucial for maintaining health. While bacteria dominate this system, fungal communities, collectively called the mycobiome, are increasingly recognized as vital contributors. However, fungi remain understudied due to challenges in culturing many species, limiting our understanding of their roles, interactions and effects on human biology. Advances in next-generation sequencing have transformed mycobiome research, revealing fungal diversity and its impact on health and disease. This review examines the mycobiome’s composition and function across major body sites, including the gut, mouth, lungs, reproductive tract and skin. It also explores connections between fungal imbalances (dysbiosis) and diseases such as neurological disorders, cancer and post-COVID-19 complications. Despite progress, challenges persist, including the need for better culture-independent diagnostic tools and standardized research methods. Combining culturomics and metagenomics could help overcome these limitations and identify new treatment targets. By summarizing current knowledge and highlighting research gaps, this review aims to guide future studies on the mycobiome’s role in human health.

## Introduction

The human body harbours a complex and dynamic microbial community, consisting of both cellular micro-organisms (including bacteria, fungi and protozoa) and non-cellular entities (such as viruses), collectively referred to as the microbiome. This microbial community maintains a highly intricate and symbiotic relationship with its host, the human body [1,3]. Bacteria represent over 99% of the microbial population in humans and other organisms, forming the core microbiome. In contrast, fungi, though less abundant and diverse than bacteria, constitute a distinct subset known as the mycobiome [4]. The term human mycobiome was first introduced by Ghannoum *et al*. in 2010 to describe the fungal communities present in the oral cavities of healthy individuals [5]. Since then, scientific research on the mycobiome has significantly expanded, with a growing number of publications addressing this topic each year [6,9]. Fungi begin colonizing various parts of the body, such as the skin, oral and vaginal mucosa, gastrointestinal and respiratory tracts and genitourinary system, immediately after birth [10]. The initial composition of an infant’s mycobiome is shaped by factors including gestational age, birth weight, mode of delivery and feeding practices [11,12]. Passage through the birth canal represents an infant’s first major contact with microbes, initiating colonization. While the microbiome of healthy newborns is not yet fully understood, research has emphasized fungal colonization in premature infants. Those born very early and weighing less than 1,500 g are at high risk of invasive *Candida* infections. Their vulnerability is linked not only to gut and immune immaturity but also to factors such as early antibiotic exposure, higher caesarean rates, delayed feeding and evidence that more than half develop gut *Candida* colonization within 6 weeks [13]. Over time, numerous internal and external influences, such as diet, body weight, age, gender, exposure to antibacterial and antifungal treatments and environmental factors, contribute to alterations in the mycobiome [11,12].

Diet significantly influences the gut microbiome, as foodborne fungi are present in both plant and animal foods. Research has shown that *Saccharomyces* and *Hannaella* are linked to butter and animal fats, whereas *Aspergillus* is related to eggs and processed legumes. Diverging trends are noted with whole grains, fish and seafood. Notably, *Fusarium* is prevalent among vegetarians (88%) but rare in omnivores (3%) [10]. In addition to diet, a significant reduction in biodiversity was observed in overweight and obese individuals compared to healthy individuals adhering to a eutrophic diet, respectively. An in-depth analysis of a young woman with anorexia nervosa revealed specific mycobiome species, such as *Penicillium solitum* and *Cladosporium bruhnei* [14]. Strati *et al*. [15] discovered that the gut mycobiome is affected by both age and sex. They investigated the species diversity of the gut mycobiome in infants and children versus adults using culture-independent methods. The research showed no notable differences linked to age. Nonetheless, notable sex-related differences were noted, with females exhibiting a greater number of isolates and documented species than males. The genera *Penicillium*, *Aspergillus* and *Candida* were common among their subgroup of healthy individuals [15]. Regarding the impact of antibiotics and antifungals on the mycobiome, we can discuss a condition called vulvovaginal candidiasis (VVC), where elevated antibiotic consumption leads to a reduction of beneficial bacteria such as lactobacilli (which are integral to the healthy vaginal microbiome) and an excessive growth of *Candida* species [16]. An important aspect of many studies is the impact of environmental factors, which can significantly influence the human mycobiome. These include smoking, alcohol consumption and eating disorders (EDs) such as anorexia nervosa and bulimia nervosa, among other conditions, all of which have been researched in relation to the mycobiome, especially the human gut and oral mycobiome [17]. This subject is examined more thoroughly in other sections.

Although fungi make up only a small fraction of the human microbiome, they play a vital role in maintaining health and are implicated in various diseases [18]. Over the past few decades, the incidence of fungal infections, particularly opportunistic ones, has risen sharply, especially among immunosuppressed individuals, such as those with AIDS, organ transplant recipients and patients undergoing chemotherapy for cancer [19]. Additionally, research has identified correlations between specific mycobiomes and various diseases, including hepatitis B [20], cystic fibrosis (CF) [21,22] and inflammatory bowel disease (IBD) [23]. The dynamic interactions within microbial ecosystems, as well as between the mycobiome and its host, play a crucial role in maintaining health or contributing to disease progression [24]. The growing recognition of fungi’s significance in human health and disease has fueled increased research interest in the mycobiome [25]. However, a comprehensive assessment of the most effective methodologies for studying and characterizing the human mycobiome remains lacking [17]. The choice of analytical approach significantly impacts research outcomes and interpretations [26]. Traditional culture-based techniques, which involve isolating and cultivating fungi, enable detailed analysis of the most abundant and readily culturable fungal species [27]. However, given the high proportion of fungi that cannot be cultivated under standard laboratory conditions, reliance on culture-based methods alone fails to provide an accurate representation of the fungal community. Consequently, this limitation restricts a deeper and more precise understanding of fungal microbiota [17]. To overcome these limitations, significant advancements in molecular techniques, particularly high-throughput sequencing methods such as next-generation sequencing (NGS) platforms, have been developed. These technologies have greatly improved our ability to analyse the complexity of fungal communities across different regions of the human body, providing deeper insights into the role of fungi in health and disease [17,28]. In this review, we will provide a comprehensive examination of the human mycobiome, covering its presence in the gut, oral cavity, respiratory tract, genital tract and skin. Additionally, we will explore the associations between the mycobiome and neurological disorders, cancer and alterations observed in coronavirus disease 2019 (COVID-19) patients. Furthermore, recent advancements and diagnostic challenges related to mycobiome research will be discussed, offering new perspectives and valuable guidance for future studies in this field.

## Gut mycobiome

The gut hosts the largest and most diverse segment of the human microbiome, consisting of an estimated one trillion micro-organisms [29]. While extensive research has been conducted on the bacterial components of the gut microbiome, the diversity and functional roles of gut-associated fungi remain relatively unexplored, lagging significantly behind other areas of microbiome research [13].

Fungi constitute a small proportion, ~0.1%, of the total microbial population in the gut [30]. The predominant fungal groups within the gut mycobiome belong primarily to the phylum Ascomycota, followed by Zygomycota and Basidiomycota [31,32]. Common genera include *Candida*, *Cryptococcus*, *Malassezia*, *Aspergillus*, *Saccharomyces*, *Galactomyces*, *Trichosporon* and *Cladosporium* [33]. *Candida albicans* is a prevalent fungal pathogen in humans, causing millions of symptomatic infections each year [34]. Humans usually become colonized by this fungus during childhood, where clonal fungal populations remain as asymptomatic commensals; however, it can lead to pathologies often associated with immunodeficiencies, varying from mild irritations to severe invasive infections [35]. A crucial factor in its ability to cause disease is the belief that fungal spread resulting in systemic infection starts from the gut, its main reservoir as an innocuous commensal [36]. Commensal fungi, especially *C. albicans*, serve not only as pathogens but also as significant regulators of the myeloid innate immune system, initiating both cellular and humoral immune responses. This highlights the essential importance of comprehending their interactions with the host and various micro-organisms for the overall physiology of the host [37,38]. Kralova *et al*. [39] recently identified a new fungal commensal, *Kazachstania heterogenica* var. *weizmannii*, in the murine intestines. Significantly, exposure to *K. weizmannii* was found to inhibit *C. albicans* colonization and drastically reduce existing loads in already colonized mice. This competitive fungal commensalism effectively reduced fatal candidiasis following immunosuppression, a finding with potential human significance, as metagenome studies have detected related species in human commensals. Consequently, their findings reveal a form of inter-fungal competition within the gut microbiota that operates independently of bacteria and the host immune response, highlighting its significant therapeutic promise for managing conditions associated with *C. albicans* [39].

Studies have shown that neonates delivered vaginally have markedly higher rates of fungal colonization than those born by caesarean section (C-section) [40]. Vaginally delivered infants are more likely to acquire fungal species from the maternal vaginal microbiota, while those delivered through C-section primarily harbour fungi associated with the maternal skin [11,41]. Fungal colonization in the infant gut can occur through vertical transmission (directly from mother to child) immediately after birth or through horizontal transmission (acquired from the environment) over time [11]. Another potential source of fungal colonization is breast milk, as the diversity of the mother’s diet, particularly high-fat diets and probiotic supplementation, can influence the composition of the infant’s mycobiome [42,43]. Schei *et al*. [44] conducted a longitudinal study of 298 mother–infant pairs, showing that maternal probiotic intake during late pregnancy and breastfeeding significantly shaped gut mycobiota. Infant fungal profiles increasingly resembled maternal ones, with *Debaryomyces hansenii* dominant during breastfeeding and *Saccharomyces cerevisiae* after weaning. Probiotics boosted maternal fungi, supporting vertical fungal transmission [44].

The lower fungal diversity observed in infants compared to adults may be attributed to their young age, as their gut microbiome is still developing and has yet to be fully colonized by a wide range of fungal species [45]. The gut mycobiome has been associated with various gastrointestinal disorders, including IBD [30]. IBD encompasses a group of chronic inflammatory conditions affecting the digestive tract, primarily Crohn’s disease (CD) and ulcerative colitis. Although the precise causes of IBD remain unclear, research suggests a potential connection between alterations in the gut mycobiome and the onset of these diseases [24,30, 46]. For instance, fungal dysbiosis, an imbalance in fungal populations, has been linked to mucosal inflammation in CD patients [47]. Moreover, studies examining both bacterial and fungal microbiota have highlighted cross-kingdom interactions between fungi and bacteria that may contribute to IBD pathogenesis [48]. As a result, targeting and modulating the gut mycobiome has been proposed as a promising therapeutic approach for managing IBD [49]. Additionally, infectious viruses such as the hepatitis B virus (HBV), which impact host immunity, may exacerbate the influence of other gut microbes, including fungi, further contributing to disease progression [30]. A study using culture-independent techniques explored the relationship between fungi in the gastrointestinal tract and different stages of chronic hepatitis B infection. The research found that patients with hepatitis B-associated liver cirrhosis had significantly higher fungal species richness in their gut compared to individuals with chronic hepatitis B without liver cirrhosis. However, only minimal differences were observed in the fungal diversity of the gut between HBV-positive participants and healthy controls. These results suggest that the diversity of gut fungi is positively correlated with the progression and severity of the disease in patients at different stages of chronic hepatitis B [20].

## Oral mycobiome

The oral mycobiome is one of the most complex and diverse microbial ecosystems in the human body and has been extensively studied and characterized [10]. Fungal colonization of the oral cavity begins at birth and continues to evolve with age [50]. In adults, the oral mycobiome consists of fungi from various phyla, including Ascomycota, Basidiomycota, Glomeromycota and Mucoromycota. To date, 81 genera and 101 species of fungi from these four phyla have been identified in the oral cavity [5,51]. The most common oral fungi from Ascomycota include the yeast-like genus *Candida*, while Basidiomycota species such as *Rhodotorula*, *Malassezia* and *Cryptococcus* are also prevalent [5,52]. The most frequently found oral fungi are yeasts of the *Candida* genus, with their abundance varying according to age. The prevalence of *Candida* is lower in infants compared to 1-year-old children [25]. Additionally, neonates with very low birth weight demonstrated higher colonization rates with non-*albicans Candida* (NAC) species compared to those weighing >1,500 g, who primarily showed *C. albicans* colonization [53]. Research has shown a strong correlation between the composition of the oral mycobiome and factors such as advancing age, tooth loss, reduced saliva production, dental caries, dietary habits, poor oral hygiene, stress and medication exposure [51,54, 55]. For example, sequencing analysis of fungal ITS1 in saliva samples from community-dwelling older adults (ages 77–99) revealed strong positive correlations between oral mycobiota composition and several age-related factors: increasing age, tooth loss (edentulism), denture usage and reduced salivary flow rates. The artificial retention niches established by dentures are likely a major contributing factor; however, other age-related physiological changes, especially reduced saliva production and changes in the mucosa, seem to significantly affect the dynamic restructuring of the oral mycobiome [56].

Geographical variations have been suggested to influence the composition of the oral mycobiome. For example, a study found that the prevalence of *Candida* in healthy individuals was lower in Asia compared to Western countries [57]. In addition to geographical factors, habitual behaviours also play a significant role in shaping the oral mycobiome [17]. Habits such as smoking and EDs like anorexia nervosa and bulimia nervosa have been shown to impact fungal communities in the oral cavity [58,59]. One study revealed that smokers had higher levels of yeast and harmful moulds in their oral microbiome compared to non-smokers. Researchers have suggested that the changes in the composition of the oral mycobiome caused by tobacco smoking could contribute to the development of oral cancer (OC) by increasing the presence of pathogenic fungi, which can lead to opportunistic infections [60]. Table 1 summarizes the prevalence of fungal species in the oral mycobiome of healthy individuals and patients with various oral pathologies.

**Table 1.** Prevalence of fungal species in the oral mycobiome of healthy individuals and patients with various oral pathologies

Healthy status			Disease status			
Genus	Species	Citation	Various oral pathologies	Genus	Species	Citation
*Candida*	*C. albicans*, *C. dubliniensis*, *C. guilliermondii*, *C. khmerensis, C. metapsilosis*, *C. parapsilosis*, *C. tropicalis*, *C. quercitrusa*, *C. sake*	[5,51, 165,167]	Periodontal	*Candida*	*C. albicans*, *C. dubliniensis*, *C. parapsilosis*, *C. quercitrusa*, *C. sake*, *C. tropicalis*	[51]
				*Aspergillus*	*A. niger*	[51]
*Saccharomyces*	*S. cerevisiae*, *S. bayanus*, *S. ellipsoideus*	[5,165]	Caries	*Candida*	*C. albicans*, *C. sake*	[168,169]
				*Cryptococcus*	*C. neoformans*	
				*Malassezia*	*M. globosa*	
*Alternaria*	*A. tenuissima*, *A. triticina*, *A. infectoria*	[5,167]	Peri-implantitis	*Aspergillus*	*A. restrictus*	[170]
*Aspergillus*	*A. amstelodami*, *A. caesiellus*, *A. flavus*, *A. oryzae*, *A. penicillioides*, *A. ruber*, *A. nidulans*, *A. niger*	[5,166, 167]	Oral lichen planus	*Candida*	*C. albicans*, *C. dubliniensis*, *C. glabrata*, *C. krusei*, *C. parapsilosis*	[171,172]
				*Cladosporium*	*C. cladosporioides*, *C. herbarum*, *C. sphaerospermum*, *C. tenuissimum*	[5,166]	Leukoplakia	*Candida*	*C. albicans*	[173]
*Cryptococcus*	*C. cellulolyticus*, *C. diffluens*	[5]	OC	*Candida*	*C. albicans*, *C. glabarata*, *C. kruseii*, *C. tropicalis*, *C. parapsilosis*, *C. dubliniensis*	[174,178]
*Fusarium*	*F. culmorum*, *F. oxysporum*, *F. poae*	[5,167]		*Verruconis*	*V. gallopava*	[179]
*Glomus*	*G. fulvum*, *G. mosseae*	[5]		*Syncephalastrum*	*S. racemosum*	
*Ophiostoma*	*O. floccosum*, *O. pulvinisporum*	[5]		*Dimargaris*	*D. cristalligena*	
*Penicillium*	*P. brevicompactum*, *P. glabrum*, *P. spinulosum*	[5,166]		*Lichtheimia*	*L.corymbifera*	
*Phoma*	*P. foveata*, *P. plurivora*	[5]		*Malassezia*	*M. sympodialis*	
*Schizosaccharomyces*	*S. japonicus*, *S. pombe*	[5]		*Acremonium*	*A. exuviarum*	[180]
*Zygosaccharomyces*	*Z. pseudorouxii*, *Z. rouxii*	[5]		*Aspergillus*	*A. fumigatus*	
*Rhodotorula*		[166]		*Morchella*	*M. septimelata*	
*Pichia*	*P. jadinii*, *P. kudriavzevii*, *P. membranifaciens*	[167]		*Mortierella*	*M. echinula*	
*Filobasidium*	*F. floriforme*	[167]		*Schizophyllum*	*S. commun*	[181]
*Cystofilobasidium*	*C. macerans*	[167]		*Cyberlindnera*		[146]
*Pleosporales*		[51]	Recurrent aphthous stomatitis	*Malassezia* and *Candida*	*C. albicans*	[182]
*Kluyveromyces*	*K. lactis*	[165]				

## Respiratory tract mycobiome

Historically, the lungs of healthy individuals were believed to be sterile. However, recent studies have shown that the respiratory tract, including the lungs, harbours a diverse microbiota that plays a role in various diseases [61,62]. The composition of the lung mycobiome is likely unique to each individual [63,64]. In healthy individuals, fungal spores that are inhaled from the environment into the respiratory tract are typically cleared efficiently through mucociliary action and phagocytosis [65,66]. In contrast, individuals with compromised immune systems or underlying conditions, such as neutropenia, asthma or lung diseases, struggle to clear fungal spores effectively. As a result, these spores can persist, colonize the lungs and contribute to the development of respiratory diseases [66]. Despite limited research on the respiratory tract mycobiome, some studies have identified the presence of fungal species from the Ascomycota and Basidiomycota phyla in different regions of the respiratory tract [67,68]. For example, *Cryptococcus magnus* from the Basidiomycota phylum has been identified in the lungs [68]. Other species, including *Peniophora incarnata*, *Peniophora cinerea*, *Daedaleopsis confragosa*, *Sistotrema brinkmannii* and *Stereum hirsutum*, which are known phytopathogens, have also been detected. This suggests that these species may enter the airways through microaspiration and are not permanent residents of the respiratory tract [10,68]. Significantly, several of these fungi, including ~50% of those linked to marijuana smoking, are currently classified as phytopathogens yet demonstrate opportunistic microbial potential in humans. Individuals who smoke marijuana may exhibit increased susceptibility to colonization by these plant-associated fungi, although their clinical implications for respiratory health remain incompletely characterized [68]. A clinically relevant example is *Ceriporia lacerata*, currently recognized as an opportunistic basidiomycetous pathogen capable of inducing pneumonia, despite its original classification as solely responsible for white-rot wood decay [69]. Similarly, established phytopathogens, including *Aspergillus flavus*, demonstrate significant zoonotic potential, capable of progressing to fatal systemic infections in both animal hosts and human populations [70].

The mycobiome of healthy lungs differs from that found in individuals with chronic inflammatory respiratory conditions, such as asthma, chronic obstructive pulmonary disease (COPD), CF and bronchiectasis. In these patients, impaired lung function can lead to fungal overgrowth and/or the loss of specific fungal species, resulting in reduced fungal diversity [71]. For example, in asthma patients, there is a higher prevalence of species like *Psathyrella candolleana*, *Malassezia pachydermatis*, *Termitomyces clypeatus* and *Grifola sordulenta*, along with greater fungal diversity compared to healthy individuals [18,71]. Children with severe asthma have shown an increased frequency of genera such as *Rhodosporidium*, *Pneumocystis*, *Leucosporidium* and *Rhodotorula* when compared to healthy children. Additionally, patients with CF exhibit a higher abundance of *Malassezia*, *Candida* and *Aspergillus* species [72]. In COPD patients, *Aspergillus* species have been isolated in 17% of cases [73]. *Aspergillus* spores are known to play a role in allergic respiratory conditions, such as allergic rhinitis and asthma, by impacting the airways [73]. The use of antibiotics has also been identified as a contributing factor in the increased incidence of asthma and allergies, facilitating the overgrowth of commensal fungi [74].

## Vaginal mycobiome

The vagina of women of reproductive age sustains a strongly acidic environment (pH ≤4.5), mainly from lactic acid generated by native bacteria in this anaerobic setting [75]. The vaginal microbial community is additionally moulded by hormonal effects, such as cyclical epithelial shedding, glycogen metabolism via human α-amylases and innate immune superiority over adaptive responses [76]. These physiological characteristics together regulate microbial colonization of the vaginal mucosa [75].

The vaginal fungal community was first recognized by Castellani in 1929 [77], and since then, fungi have been increasingly recognized for their role as a key component of the vaginal microbiota in healthy women. The composition of the vaginal mycobiome is influenced by factors such as diet, environmental exposures and lifestyle choices. Similar to other body sites, the initial composition of the vaginal mycobiome is affected by the mode of delivery and the conditions at birth [41,78]. Over time, the vaginal mycobiome evolves, particularly during the pre-pubertal, post-pubertal and menstrual phases, and further changes occur during pregnancy [79].

The vaginal mycobiome is primarily composed of yeast-like micro-organisms from the phylum Ascomycota, with at least 22 fungal genera naturally found in the vaginal environment of healthy women [75]. The genus *Candida*, especially *C. albicans*, is the most common and prevalent component of the vaginal mycobiome in healthy women [80]. *C. albicans* is the primary cause of VVC, affecting about 75% of women of reproductive age, with ~7% experiencing recurrent episodes (≥4 per year) [81,82]. Symptoms such as itching, burning, discharge and dyspareunia result mainly from a hyperinflammatory response to *C. albicans*, as the fungus can colonize asymptomatically [83]. VVC arises from multiple factors, including disrupted vaginal microbiota, host genetic susceptibility and *Candida* virulence traits [84]. Current management for recurrent cases involves 6 months of azole therapy (e.g. fluconazole) [83], but over half of patients relapse within 6 months after stopping treatment, partly due to rising azole resistance in clinical isolates [85]. Immunologically, VVC is linked to S100A8/A9 alarmins that drive vaginal inflammation and recruit nonprotective neutrophils [86,87], though no fungal virulence factor has been definitively tied to human immunopathology. Notably, S100A8/A9 elevates expression of *C. albicans* PRA1 [88], a pH-regulated secreted protein influencing complement activity, neutrophil chemotaxis and immune dysregulation [89]. Roselletti *et al*. [90] identified PRA1 as a major immunopathological factor in VVC: it is strongly upregulated during infection, correlates with inflammatory cytokines and promotes vaginal inflammation. PRA1 deletion or zinc-mediated inhibition alleviated disease in mice, while zinc gel therapy reduced recurrent VVC in women, positioning PRA1 as a promising target for future preventive treatments [90].

Among NAC species, *C. glabrata* is the most frequently reported [80], representing around 10–20% of occurrences, followed by *C. parapsilosis*, *C. tropicalis*, *C. krusei*, *C. africana*, *C. dubliniensis* and *C. guilliermondii* [91,93]. The incidence and distribution of NAC species vary geographically and across populations, with research indicating a significant increase in NAC-associated VVC cases [94]. Regions such as Tunisia, Nigeria, Middle Eastern countries and Asia have reported particularly high prevalence rates, where *C. glabrata* constitutes 30–50% of isolated NAC species [84]. Furthermore, NAC infections are associated with a higher tendency for recurrence in VVC patients [95], likely due to their intrinsic reduced susceptibility to conventional azole therapy [96]. Besides *Candida* species, *S. cerevisiae* is also recognized as part of the vaginal mycobiome [91].

## Skin mycobiome

**Fig. 1.**
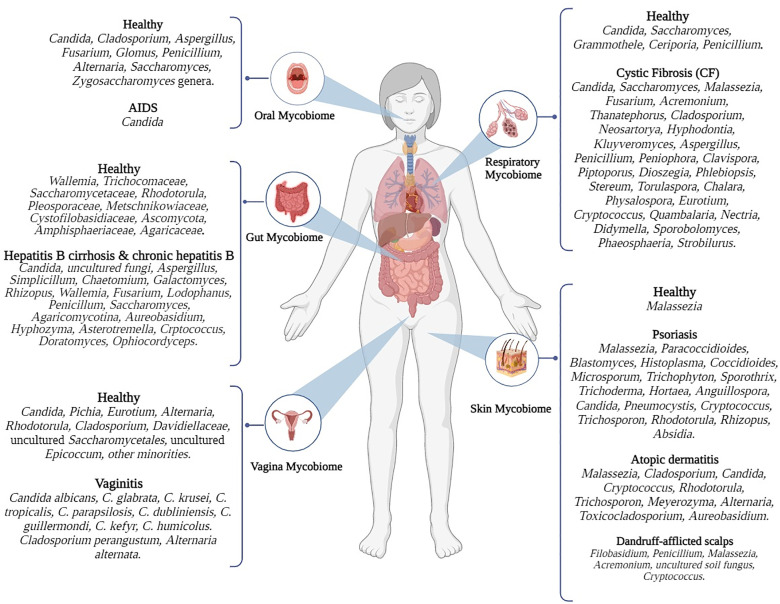
Summary of mycobiomes in health or disease status in different body sites.

The skin, the largest organ in the human body, hosts a diverse range of microbial species [18]. Fungi make up less than 10% of the total microbial population on the skin [7], with at least 168 genera identified, predominantly from the Basidiomycota and Ascomycota phyla [97]. Yeasts from the *Malassezia* genus are the most common fungal residents, with higher concentrations in areas like the elbows, back, outer ear canal, nostrils and various facial regions [98,99]. *Malassezia* produces an aryl hydrocarbon receptor ligand that aids in maintaining epithelial cell health and provides UV protection, which is beneficial for overall skin health [98]. Another Basidiomycota genus, *Cryptococcus*, has also been found on the skin of healthy individuals. Other genera, including *Aspergillus*, *Penicillium* and *Epicoccum*, are rarely detected [97,98, 100]. The skin mycobiome varies with age, with *Aspergillus*, *Epicoccum*, *Phoma* and *Malassezia globosa* being common in pre-pubertal individuals, while *M. restricta* predominates in the post-pubertal period. Gender differences also influence the mycobiome composition; for instance, adolescent boys tend to have higher levels of *Epicococcomus* and *Cryptococcus*, while *Malassezia* is more commonly found in girls [101]. Fig. 1 provides an overview of mycobiomes associated with various body sites in both healthy and diseased states.

## Mycobiome and neurological disorders

Recent research has increasingly focused on the connection between the mycobiome and central nervous system disorders. Studies suggest that changes in gut microbiota, including fungi, are associated with the development of conditions like multiple sclerosis (MS), emphasizing the role of gut-brain interactions [102,104]. In individuals with MS, targeted internal transcribed spacer (ITS)-1 sequencing has revealed elevated levels of *Candida*, *Malassezia* and *Trichosporon* in brain tissue [103]. Furthermore, metagenomic analyses of cerebrospinal fluid (CSF) from MS patients identified the presence of *Malassezia*, *Ascomycota*, *Funneliformis*, *Glomus*, *Cladosporium*, *Candida* and *Alternaria*, though some fungi may have been environmental contaminants [105]. Additionally, a study on Alzheimer’s disease patients found fungal cells within neurons, identified using immunohistochemistry and confocal microscopy techniques. The fungal species detected, primarily from the genera *Candida*, *Cladosporium*, *Malassezia*, *Neosartorya*, *Phoma* and *Saccharomyces*, were further confirmed by nested PCR [106]. Targeted amplicon sequencing also showed a higher prevalence of *Alternaria* and *Malassezia* in the brain tissue of Alzheimer’s patients compared to healthy controls [107]. Another investigation revealed a higher prevalence of *Candida*, *Malassezia*, *Fusarium*, *Botrytis*, *Trichoderma* and *Cryptococcus* genera in the brain tissue of amyotrophic lateral sclerosis (ALS) patients [108]. Additionally, recent research has suggested a significant link between seborrhoeic dermatitis and Parkinson’s disease, proposing that *Malassezia* might contribute to the development of both conditions [109].

## Mycobiome and cancer

Head and neck cancers are the sixth most common cancer globally, with OC and oropharyngeal carcinoma being the most prevalent forms [110]. Changes in the oral microbiome and mycobiome may contribute to carcinogenesis through the metabolites produced by micro-organisms. For example, acetaldehyde, a byproduct of alcohol metabolism, has been associated with OC, particularly in chronic alcohol users. The presence of bacteria and fungi, such as *Rothia*, *Streptococcus*, *Prevotella* [111] and *C. albicans* [112], which also produce acetaldehyde, may play a role in oral tumour development. In a study by Perera *et al*., *C. albicans* was found to be more abundant in oral squamous cell carcinoma tissue compared to benign tissue (intra-oral fibro-epithelial polyps) [113]. Additionally, some studies suggest that *C. albicans* may induce inflammation and carcinogenesis by promoting hypermethylation of various tumour suppressor genes [114]. Beyond *C. albicans*, *Schizophyllum commune*, a member of the Basidiomycota phylum, was frequently detected in the oral wash of healthy individuals [110]. This fungal species is notable for its production of schizophyllan (SPG), a polysaccharide with antitumour properties [115]. In studies on animals, SPG exhibited notable immunomodulatory properties, providing defence against bacterial infections by enhancing the host immune response [116]. The compound’s antitumour properties were initially documented in various murine models, including transplanted tumour systems [117] and Lewis lung carcinoma (a well-established metastasis model) [118], with subsequent confirmation in rat tumour models [119]. SPG has also been noted to display T-cell adjuvant effects [120].

## Mycobiome in patients with COVID-19

COVID-19 has impacted over 100 million individuals globally [121,123]. Recent research has highlighted that immune dysregulation is closely associated with COVID-19 infection [124]. The gut mycobiome is essential for maintaining immune homeostasis and protecting against infections, and its dysregulation in the gastrointestinal tract is a hallmark of several diseases associated with systemic effects [125]. For instance, patients with COVID-19 have been shown to exhibit alterations in their gut mycobiome, including an increase in *C. albicans* and other diverse fungal species. The proliferation of opportunistic fungi, such as *A. flavus*, *C. albicans* and *C. auris*, has also been documented in stool samples from COVID-19 patients [126]. These changes in the gut mycobiome may contribute to the immune system dysfunction seen in COVID-19 patients. Kusakabe *et al*. [127] reported that elevated *C. albicans* IgG antibodies serve as a marker in severe COVID-19 (sCOVID-19) patients, who also displayed intestinal *Candida* overgrowth, mycobiota dysbiosis and systemic neutrophilia. Analysis of haematopoietic stem cell progenitors revealed altered expression of antifungal immunity–related genes and long-term reprogramming of granulocyte myeloid progenitors (GMPs), persisting for up to a year. In mice, infection with patient-derived *C. albicans* strains enhanced lung neutrophilia and pulmonary NETosis during SARS-CoV-2 infection, effects that were partially reduced by antifungal therapy or IL-6 receptor blockade. Consistently, COVID-19 patients treated with tocilizumab exhibited reduced *C. albicans* IgG levels and reversal of GMP transcriptional changes. These findings suggest that intestinal fungal pathobionts intensify immune activation in sCOVID-19 and highlight the potential of targeting the mycobiota–immune axis as a therapeutic strategy for patients with prolonged symptoms [127].

Maeda *et al*. [128] noted that it remains unclear whether alterations in mycobiome composition act as a predisposing factor for COVID-19 infection or arise as a consequence of the virus-induced inflammatory response [128]. ACE-2 receptors, found on cells in the intestinal epithelium, oesophagus, liver and lungs, facilitate viral entry and infection. During fermentation, microbiota metabolites may block the ACE-2 receptor or viral proteins, potentially preventing viral spread [129]. A study conducted in Japan explored the mycobiome and microbiome across three groups: individuals with sCOVID-19, those with mild COVID-19 and healthy controls. Both severe and mild COVID-19 patients exhibited a reduction in fungal diversity. In certain cases, *Candida* species, particularly *C. albicans*, were dominant in the mycobiome, with sCOVID-19 patients showing a higher prevalence of opportunistic bacteria such as *Enterococcus*, *Alistipes* and *Lactobacillus* [128]. Similarly, a study in China observed changes in the mycobiome composition of COVID-19 patients, marked by a decrease in fungal diversity and an increase in *C. albicans*, *C. auris* and *A. flavus* [126]. These changes in the gut mycobiome and microbiome of COVID-19 patients can persist for at least 6 months, likely due to prolonged inflammation. The persistence of symptoms associated with COVID-19, referred to as ‘long COVID’, may involve ongoing alterations in the mycobiome and gut microbiota. The use of antifungal treatments, such as fluconazole, may also contribute to changes in the gut mycobiome. Research suggests that the mycobiome could serve as a more sensitive biomarker than the microbiota for assessing the severity of COVID-19 [128]. Comparing studies is difficult due to variations in patient clinical conditions, such as the use of antibiotics and immunosuppressive drugs, as well as differences in disease severity. The gut mycobiota and microbiota influence host immune responses, which may play a role in the persistence of long-term symptoms associated with COVID-19 [128,130]. Although more detailed studies are necessary to further understand the relationship between the mycobiome and microbiome, recent research indicates that targeting the gut microbiota in patients recovering from sCOVID-19 could help alleviate lingering symptoms of the disease [128]. A list of selected mycobiome colonizers associated with neurological disorders, OC and COVID-19 in both health and disease states is provided in Table 2.

**Table 2.** The list of chosen neurological disorders, OC and COVID-19 mycobiome colonizers in health or disease status

Body site/identification method	Healthy or disease status	Fungal composition of the mycobiome	Citation
**Brain tissue**			
Small RNA sequencing	Healthy	*Botrytis* and *Rhodotorula*	[183]
ITS1 sequencing	MS	*Candida*, *Malassezia *and *Trichosporon*	[103]
Targeted amplicon sequencing	Alzheimer’s	*Alternaria *and *Malassezia*	[107]
Nested PCR	Alzheimer’s	*Candida*, *Cladosporium*, *Malassezia*, *Neosartorya*, *Phoma* and *Saccharomyces*	[106]
Metagenomic analysis	ALS	*Candida*, *Malassezia*, *Fusarium*, *Botrytis*, *Trichoderma* and *Cryptococcus*	[108]
**CSF**			
NGS	Healthy	Not available	[105]
	MS	*Malassezia*, *Ascomycota*, *Funneliformis*, *Glomus*, *Cladosporium*, *Candida* and *Alternaria* Bacteria: *Pseudomonas*, *Escherichia*, *Bacillus*, *Streptococcus*, *Acinetobacter*, *Corynebacterium* and *Moraxella* Parasitic/protozoan: *Albugo*, *Besnoitia*, *Babesia* and *Plasmodium*	
**Oral**			
Pyrosequencing (NGS platforms)	Healthy	*Candida* species, *Cladosporium*, *Aureobasidium*, *Saccharomycetales*, *Aspergillus*, *Fusarium and Cryptococcus*	[5]
Northern blotting and GC	Oral squamous cell carcinoma	*C. albicans*	[112] [111]
**Gut** **(faecal samples)**			
Shotgun metagenomic sequencing	Healthy	Not available	[126]
	COVID-19	*A. flavus*, *C. albicans* and *C. auris*	
**Gut** **(faecal samples)**			
Metagenomic sequencing (fungal ITS1 and bacterial 16S)	Healthy	*Aspergillus* Bacteria: *Bacteroides*, *Faecalibacterium* and *Blautia*	[128]
	COVID-19	*C. albicans* Bacteria: *Enterococcus*, *Alistipes* and *Lactobacillus*	

## Methodologies to study the human mycobiome

Studying the microbiome and mycobiome begins with sample collection, a step that poses specific challenges since fungi are typically less abundant than bacteria. When using culture-independent approaches, host cells of human or animal origin may enter the samples and interfere with the amplification and sequencing of fungal molecular markers such as rRNA genes. In addition, obtaining high-quality fungal genetic material is more demanding compared to bacterial or animal sources, making careful planning of sampling strategies essential [13].

Although high-throughput amplicon sequencing has largely replaced culture-based techniques, mycobiome research still faces significant technical hurdles. Commonly used approaches, such as sequencing of ITS1, ITS2 and 18S rRNA regions, often suffer from limitations, including inconsistent primer performance, unintended amplification of non-fungal eukaryotes and inadequate universal coverage [26]. Moreover, variation in the ITS region length and differences in tandem repeat numbers across fungal species can distort estimates of relative abundance [131]. The choice of reference database may further introduce bias, particularly when taxa lack stable classification between their teleomorph and anamorph states [17]. These constraints result in inconsistent results among studies, particularly at the genus level, and varying methodologies can yield different diversity profiles [10]. Consequently, additional research employing both integrated culture-dependent and culture-independent methods on extensive, varied cohorts from various geographical areas and health conditions is necessary. This will aid in pinpointing a core mycobiota and elucidating the mycobiome’s function in health and disease [17].

## Culture-dependent methods

Culture-dependent techniques have traditionally been employed to identify fungi in complex microbial ecosystems. These methods include classic microbiological approaches such as microscopy, biochemical assays and cultivation on selective media. Despite the rising cost of other methods, culture-based techniques are still commonly used in many laboratories [14]. While culture remains fundamental to fungal identification, it presents several challenges. On the one hand, culture-based studies can be extremely time-consuming, often requiring prolonged incubation periods [132]. On the other hand, fungal species that do not need complex nutrients may be masked by morphologically similar, rarer species on mixed primary plates, and faster-growing species can dominate the plates, even if they are initially present in low numbers [133,134]. Furthermore, uncultivable fungi, which represent a significant portion of the human mycobiome, cannot be detected by culture-based methods [17]. Another factor that can affect the outcomes of culture-dependent studies is the impact of sample storage, such as freezing faecal samples before analysis [135]. A study on cattle showed that freezing samples at −20 °C led to a 50% reduction in fungal content. Although this has been observed in cattle, its effect on human faecal samples remains uncertain [134]. Regardless, the value of culture techniques for isolating fungi should not be underestimated, as fungi isolated from pure cultures are often further investigated using more advanced methods, such as MS and DNA-based analysis [136].

## Culture-independent methods

### Biochemical methods

In medical mycology, culture-independent approaches, such as molecular techniques, serological tests and spectroscopic methods, are becoming more commonly used for diagnosing invasive fungal infections [137,138]. One such method involves detecting β-glucan, a component found in fungal cell walls, which has demonstrated sensitivities ranging from 50% to 100% and specificities between 44% and 98% in identifying invasive fungal diseases. However, β-glucan is not specific to a particular fungal species, as it is a panfungal marker [139,140]. Another widely used marker is galactomannan, which is particularly effective in diagnosing invasive aspergillosis in patients with leukaemia or neutropenia. This marker shows sensitivity between 79% and 96% and specificities ranging from 74% to 99% [140]. Additionally, amplifying fungal-specific DNA molecules from various environments and identifying them through sequencing or cloning is one of the most effective methods for conducting both quantitative and qualitative fungal studies [29,141].

### Molecular techniques

Advancements in molecular techniques have significantly reduced the reliance on culturing fungi for ecological surveys across diverse habitats [137,138]. Techniques such as PCR, Sanger sequencing and, more recently, NGS technologies allow researchers to analyse and identify microbial communities without the need for traditional culturing methods [28,142]. Additionally, several other molecular techniques have been used to explore the mycobiome, including nested and semi-nested PCR, restriction fragment length polymorphism analysis, oligonucleotide fingerprinting of rRNA genes and denaturing gradient gel electrophoresis. While these methods offer significant advantages, they have limitations in distinguishing fungal species within larger populations [24]. As of now, no single culture-independent method serves as a definitive gold standard for studying the mycobiome.

### Direct sequencing methods

NGS has become a revolutionary diagnostic method for fungal infections, surpassing significant drawbacks of traditional techniques and tackling clinical issues associated with invasive mycoses [143]. This technology shows advanced abilities in detecting both fastidious and slow-growing pathogens, demonstrating remarkable sensitivity even in paucimicrobial samples [143,144], while also delivering improved taxonomic resolution that exceeds first-generation sequencing in terms of sensitivity, speed and accuracy [145]. Even with these advancements, fungal genomics has advanced at a slower pace compared to bacterial and viral research, as sequencing databases indicated considerable underrepresentation of fungal genomes in 2014 [146], emphasizing the necessity for broader NGS applications in mycology [147]. The NGS process starts with DNA extraction from clinical specimens, then involves PCR amplification of certain fungal genomic segments [18], but existing applications encounter significant constraints in fungal research owing to technical difficulties [148].

An assessment of the high-fidelity sequencing techniques created in the last 10 years shows that 3 main error sources have been recognized and recorded; of these, library preparation has been determined to be the leading source of error, followed by the sequencing process and DNA damage [149,151]. Concerning mistakes in library construction, DNA amplification techniques lead to errors. These methods are classified into exponential amplification (PCR) and linear amplification (RCA, transcription). In PCR, mistakes occur in every cycle and are carried over into the following cycles, while in linear amplification, mistakes are not carried over into later rounds [152,154]. As a result, mistakes in linear amplification products are independent, rendering them a better choice than PCR. Strand displacement represents a type of linear amplification [155]. Nonetheless, PCR continues to be the primary technique for library preparation and the amplification of sequencing clusters. PCR inaccuracies arise from the built-in fidelity limitations of DNA polymerase and are exacerbated by the exponential amplification process [156,157]. Mistakes related to sequencing can be categorized into eight types: (1) amplification mistakes, (2) colour or laser interference mistakes, (3) noise mistakes from cluster interference or dephasing, (4) optical replication mistakes, (5) demultiplexing mistakes, (6) leftover adapter sequence mistakes, (7) base-calling mistakes and (8) skewed mistakes from specific sequence motifs [158]. In relation to errors resulting from DNA damage, the assembly of a DNA sequencing library depends on a range of complex processes, such as DNA extraction, fragmentation, end repair and A-tailing, among others [151]. In the course of these laboratory procedures, DNA damage can arise from various operational factors, especially thermal exposure and oxidative stress induced by chemical agents [159,160]. The two most prevalent forms of DNA damage observed are (1) cytosine deamination, induced by heat and leading to C-to-T transitions, and (2) guanine oxidation (8-oxoG), which results in G-to-T transversions [154].

In total, since 2011, various sequencing techniques have emerged, utilizing two main strategic approaches, error avoidance and error correction, to reduce sequencing errors [161]. In the context of minimizing errors, recognizing particular sources of mistakes has facilitated the application of appropriate corrective actions. For example, (1) the use of high-fidelity polymerase enzymes improves sequencing precision by decreasing PCR-related errors, (2) lowering the amplification cycle count or using PCR-free library preparation methods significantly lessens error introduction [151,162] and (3) errors arising from DNA damage can be mitigated through methods like restriction enzyme digestion of affected sites or by skipping procedural steps that involve heat and oxidative agents [163]. In terms of error correction, three main approaches are employed to amend errors after sequencing: consensus sequencing, double-strand alignment and validation between paired-end reads [158]. In this context, Jia *et al*. [158] offer a comprehensive overview of the main approaches to reducing sequencing inaccuracies [158].

**Fig. 2.**
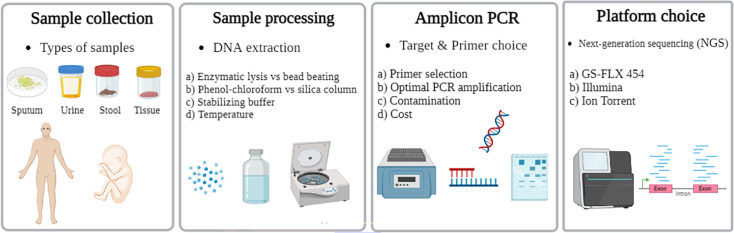
Critical steps in sequencing approaches for mycobiome investigation.

In summary, NGS acts as a powerful diagnostic tool in medical mycology because of its great sensitivity, improved resolution and ability to detect difficult-to-culture pathogens; however, its clinical applicability is limited by the inadequate representation of fungal genomes in reference databases, emphasizing the need for more extensive genomic resources. Furthermore, in spite of these analytical advantages, the widespread adoption of NGS in clinical settings faces significant challenges, especially high diagnostic costs and a widespread lack of genomics knowledge among healthcare professionals [143,164]. Key issues related to mycobiome sequencing and analysis are summarized in Fig. 2.

## Conclusion

Humans are in continuous interaction with complex microbial communities that reside across various parts of the body. Recently, there has been a growing interest in the fungal component of the human microbiome, known as the mycobiome. Although fungi are less abundant than bacteria, they exhibit considerable diversity, and our understanding of them remains in its early stages. The study of the mycobiome is challenging due to the high proportion of uncultivable fungal species that constitute a significant part of the human mycobiome. However, substantial advancements in culture-independent methods, particularly NGS, have allowed for a more detailed analysis of microbial abundance and diversity. This technology helps researchers identify specific microbial populations associated with both health and disease. Despite these advancements, there is still limited insight into the comparative advantages of different techniques for studying the fungal components of microbial communities. To move the field forward, more research is needed to develop methodological approaches, analyse sequenced isolates, establish comprehensive reference databases and conduct deeper investigations into the interactions between fungi, bacteria, viruses and the human body.

## Abbreviations

ALS: amyotrophic lateral sclerosis
CD: Crohn’s disease
CF: cystic fibrosis
COPD: chronic obstructive pulmonary disease
C-section: caesarean section
CSF: cerebrospinal fluid
EDs: eating disorders
GMPs: granulocyte myeloid progenitors
HBV: hepatitis B virus
IBD: inflammatory bowel disease
ITS: internal transcribed spacer
MS: multiple sclerosis
NAC: non-*albicans Candida*
NGS: next-generation sequencing
OC: oral cancer
sCOVID-19: severe COVID-19
SPG: schizophyllan
VVC: vulvovaginal candidiasis
